# Exercise training and de-training effects on serum leptin and TNF-α in high fat induced diabetic rats

**DOI:** 10.1186/s13098-021-00676-x

**Published:** 2021-05-26

**Authors:** Hamideh Dinari Ghozhdi, Ali Heidarianpour, Maryam Keshvari, Hassan Tavassoli

**Affiliations:** 1 Department of Exercise Physiology, Faculty of Sport Sciences, Bu-Ali Sina University, Hamedan, Iran; 2 Department of Physical Education and Sport Sciences, Faculty of Literature and Human Sciences, Lorestan University, Khorramabad, Iran

**Keywords:** Diabetes, Obesity, High-fat diet, Exercise, De-training

## Abstract

**Background:**

Adipocytokines, which are secreted by the adipose tissue, contribute to the pathogenesis of obesity-related complications. To evaluate this assumption, we investigated the effects of aerobic exercise training (AET), resistance exercise training (RET), and 4 weeks of de-training on serum leptin and TNF-α levels in diabetic rats.

**Method:**

36 Wistar rats were divided into normal diet (ND) (control, RET, AET) and high-fat diet (HFD) + STZ (control, RET, AET) groups. Serum insulin, leptin, and TNF-α levels were assessed by commercial ELISA kits. Also fasting blood glucose (FBG), low-density lipoprotein cholesterol (LDL-C), and triglycerides (TG) levels were measured by the colorimetric kits.

**Results:**

Diabetes induction increased body weight (BW) and FBG, and decreased insulin compared to the ND rats’ groups (p < 0.001). 12-weeks of AET and RET programs in the trained diabetic rats led to a decrease in TG, LDL-C, leptin, TNF-α, and FBG, and an increase in insulin compared to the HFD + STZ-C group (p < 0.001). Besides, there was no difference between AET and RET in improving the variables studied (p > 0.05). Also, de-training led to increased BW, TG, leptin, and TNF-α compared to the end of the exercise training (p < 0.05). The correlation between the variables studied was established at different stages of the study (p < 0.05), and only BW was not correlated with insulin during exercise training and de-training (p > 0.05).

**Conclusion:**

These findings indicate that both AET and RET are useful in reducing levels of serum adipocytokines (TNF-α, leptin) in diabetic and non-diabetic rats. At the same time, 4 weeks of de-training was sufficient to lose the metabolic adaptations.

## Background

Nowadays, obesity and an increase in body fat levels are among the major problems worldwide [1]. Obesity, through an inappropriate lifestyle in combination with genetic factors leads to insulin resistance, β cell stress, dysfunction of β cell and a progressive decline in insulin secretion, which eventually leads to diabetes [2, 3]. So that, in some studies, the term “Diabesity” has been used because of the strong association between obesity and diabetes [4–6]. A common risk factor in diabetes is insulin resistance caused by high-fat diet (HFD) in obese people [7]. Adipose tissue extension is also associated with enhanced adipose tissue inflammation and hypoxia, promoting recruitment of pro-inflammatory macrophages that secrete cytokines, such as tumor necrosis factor (TNF-α) and Interleukin 6 (IL-6), which, by activating the TNF-α receptor and other cytokine receptors, they worsen insulin resistance [8]. TNF-α is an important pro-inflammatory mediator that contributes to decreased expression of glucose transporter 4 (GLUT4) in adipose, skeletal, and cardiac muscle tissues leading to insulin resistance and T2DM pathogenesis [9]. In addition, TNF-α stimulates leptin mRNA expression in the adipose tissue [10]. Leptin is an adipocytokines derived from adipose tissue, which participates as an important mediator in inflammatory processes and systemic metabolism [11]. The structural and functional similarity of leptin with IL-6 has led to the leptin receptor regulates the expression of certain genes that also target IL-6 signaling, which indicates a certain amount of interaction between the two molecules [11]. Leptin and IL-6 may stimulate different aspects of bodily maintenance. While leptin appears to be involved in the regulation of T lymphocytes, IL-6 activity may specifically lead to the proliferation and differentiation of macrophages, which are involved in both inflammation and tissue repair [11]. In addition to, stimulation of leptin leads to the production of IL-6 and TNF-α in natural and laboratory conditions [11, 12], and the changes in the concentration of leptin in the cerebrospinal fluid can predict the gene expression of TNF-α in the hypothalamus [12]. Additionally, in Kupffer cells, leptin stimulates TNF-α production via the JNK and p38 MAPK pathways [13]. Due to the relationship between leptin level and C-reactive protein (CRP) (whose production is stimulated by IL-6), plasma triglycerides and fasting glucose levels [10], increased levels of free fatty acids and inflammatory cytokines increase leptin resistance (LR), which results in decreased lipid oxidation in insulin-sensitive organs, lipids, and insulin resistance [14]. Leptin has a positive relationship with adiposity, and reduced body fat leads to a reduction in its concentration. It is also involved as a metabolic hormone in regulating the hypothalamus during the exercise [15]. There was a significant relationship between better physical performance and low leptin levels in trained participants. An effective intensity training program can temporarily reduce leptin concentrations before and after exercise in humans [16]. There is currently no cure for this problem. Given the close relationship between energy consumption and energy cost (J-shaped), an increase in energy expenditure is a good approach to tackle the global obesity epidemic and type 2 diabetes mellitus (T2DM) [17]. Therefore, physical exercise may be the first and best therapeutic intervention for metabolic disorders. Studies have shown that exercise training can help glucose control, lipid profile, and abdominal adiposity and help to improve metabolic processes [18]. In recent research, we showed that exercise reduced blood glucose level, insulin resistance and the area under the curve (AUC) in the oral glucose tolerance test (OGTT) in type 2 diabetic rats [19]. Endurance exercise reduces leptin concentrations during rest and immediately after exercise and acts as an intermediary between daily energy intake and energy consumption [20]. Both TNF-α, and leptin have circadian rhythmicity in the rat brain, so both are known cytokines that converge in the literature on chronic inflammatory states. The positive relationship between leptin and TNF-R1 in obese individuals confirms that TNF-α can be involved in regulating plasma leptin concentrations [21]. De-training refers to a minor or complete reduction in training adaptation after stopping regular exercise. Two weeks of de-training, adaptations to cytokines, and cardiac function in hypertensive rats were reversed, but this time, it was not enough to eliminate the beneficial effects of regular exercise [22]. Clinically, among the metabolic abnormalities that commonly accompany diabetes are disturbances in the production and clearance of plasma lipoproteins and development of dyslipidemia. Currently, few studies have been performed to elucidate the association between metabolic parameters, leptin and TNF-α following exercise training and de-training. Using a proper model to induce T2DM in rats and then accurate monitoring of the results during different steps of the protocol can enhance our understanding of this issue. Although aerobic exercise training (AET) program response is the prevalent form of exercise used for rats, we have used it with resistance exercise training (RET) and also HFD-STZ diabetic model; because it is similar to the features of type 2 diabetes in human. This study aimed to investigate the effects of 4 weeks of HFD consumption with a low dose of STZ, 12 weeks of AET (5 days/week) and RET (3 days/week), and 4 weeks of de-training on leptin, TNF-α and other metabolic parameters in type 2 diabetic rats.

## Materials and methods

### Animals

**Fig. 1:**
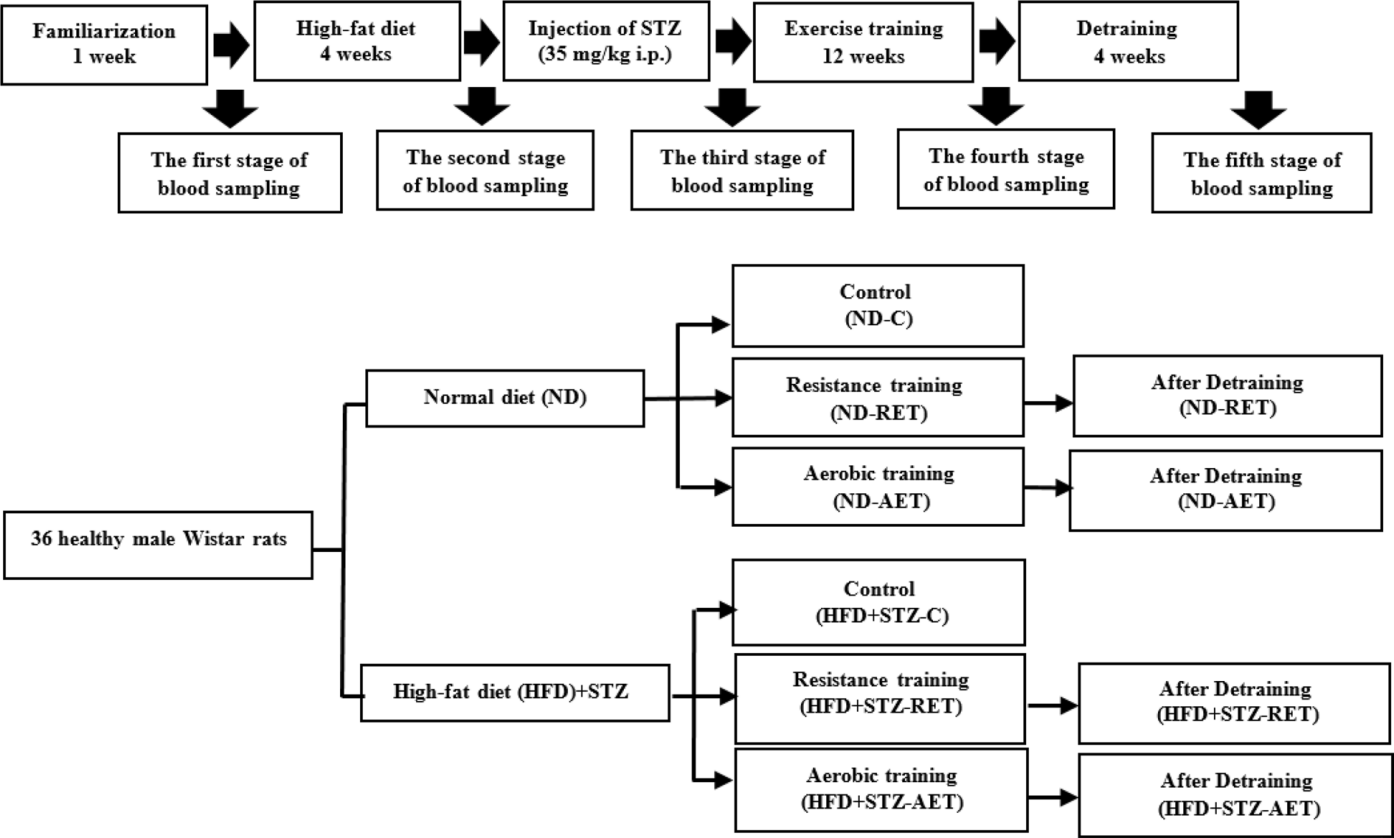
Experimental design. 36 healthy male Wistar rats were randomly divided to two main groups: (1) Healthy rats that received a normal diet (ND; n = 18) (control (ND-C), resistance exercise training (ND-RET), and aerobic exercise training (ND-AET)) (2) Low-dose STZ-induced diabetic rats that received a high-fat diet (HFD + STZ; n = 18) (control (HFD + STZ-C), resistance exercise training (HFD + STZ-RET), and aerobic exercise training (HFD + STZ-AET)). For accurate monitoring, samplings were performed in various stages (beginning, after 4 weeks of HFD, after STZ injection, after exercise training, and after de-training)

Thirty-six Wistar rats weighing 200–250 g were purchased from the Pasteur Institute of Tehran, and transferred to the animal laboratory of Bu-Ali Sina University (BASU) in Hamedan (Iran). All rats were kept in polycarbonate cages under standard conditions (22 ± 2 °C, 50 ± 5% humidity,12 h light/12 h dark cycle, respectively) with free availability of water, and food. All experimental steps were approved by the BASU Laboratory Animal Care and Use Ethics Committee. Figure 1 shows an outline of the grouping of animals and the timing of the study.

### Animal feeding

After a week of adaptation to the laboratory environment, HFD was set out for the diabetic rats’ group. The total caloric content of HFD was ∼ 4900 kcal/kg, which consisted of 27.5% carbohydrate calories, 58% fat calories and 14.5% protein calories. The method for preparing the HFD in our laboratory has been previously explained [23]. The HFD content food was ready for weekly consumption and was kept at − 4 °C. Also, the normal-pellet diet was purchased from an animal feed company called “Pars Animal Feed”. They were given free and easy access to water and food. At the same time, healthy rats had a normal diet with a total caloric content of ∼ 3160 kcal/kg. The normal diet consisted of 57% carbohydrates, 2% fat, 17.5% protein, 4.9% vitamin and mineral mix, 6.6% fiber, 12% water (see Table 1).

**Table 1:** Normal pellet diet (ND) and high fat diet (HFD) compounds [23]

	ND		HFD	
	Percent by weight	Percent by calorie	Percent by weight	Percent by calorie
Carbohydrate	57	72.1	33.3	27.5
Fat	2	5.6	32.3	58
Protein	17.5	22.1	17.5	14.5
Vitamin and mineral mix	4.9	–	5.7	–
Fiber	6.6	–	3.9	–
Water	12	–	7.1	–
Total	100	100	100	100

### Diabetes mellitus type 2 induction

Type 2 diabetes was induced by a 4-week diet with HFD followed by a single subcutaneous injection of STZ (35 mg/kg dissolved in 0.1 mM citrate buffer, pH 4.5; Sigma-Aldrich, St. Louis, Missouri, United States) in the fasting conditions. Fasting blood glucose (FBG) levels were determined by a glucometer (Acuu-Chek Active, Roche, Germany) 72 h after taking tail blood samples from the rats. The rats with FBG higher than 300 mg/dl were considered as diabetic [24], and were placed in the HFD + STZ group (HFD + STZ-C, HFD + STZ-RET, HFD + STZ-AET). According to Lee index, the rats with an index higher than 0.3 were obese [23].

### Exercise training protocol

Before the induction of diabetes in the rats, they were trained on how to climb the ladder and run on the treadmill. RET included climbing a ladder while weight was hung from the rat’s tail (3 days/week, for 12 weeks). The training method was such that in the first stage, the rat climbed the ladder without bearing additional load. To determine one-repetition maximum (1RM), in the next attempt 20 g was added to the load weight. The load continued until the rats were no longer able to continue training. The rest period between each stage was 2 min. The intensity of the main training protocol for the first to third week was equal to 50% of 1RM. Then the load was increased to 70, 80, and 100% of 1RM in the next steps, each of which lasted 3 weeks. For the warm-up and cool-down period, the rats climbed the ladder twice per session, without bearing any additional load [25].

Before starting the main aerobic program, all the rats in the aerobic exercise group were introduced to the treadmill for 7 days (5–8 m/min, 10–15 min/day) in order to be able to use it properly. Then, they performed the training protocol for 12 weeks, starting with 10 min on the first day and reaching 60 min in the last week. The average intensity of AET protocol was 26 m/min (0% grade). As a warm-up and cool-down step, the rats ran with low (10 m/min) intensities in each session. The rats in the control groups were completely sedentary [26].

### Method of blood sampling

Following 10–12 h of fasting, the rats were put into anesthesia using sodium pentobarbital and sufficient blood samples were taken from the eyes area. For accurate monitoring, samplings were performed in various stages (at the beginning, at the end of the 4th week of the HFD diet, 72 h after STZ injection, 48 h after exercise and at the end of the 4th week of de-training). To collect serum samples, blood samples were centrifuged at 2500 rpm for 15 min and stored at – 70 °C. Serum gel tubes were used to collect blood samples and microtubules were used for serum samples.

The concentrations of Leptin and TNF-α (Zellbio, GmbH, Germany- Diaclone, GmbH, and French), and serum insulin (Mercodia, Uppsala, Sweden) level were measured using ELISA method. The intra-assay coefficient of variation and sensitivity were 6.3%, 0.2 ng/Ml for leptin, 0.02 pg/Ml for TNF-α and 6.1%, 0.07 µg/l for insulin. The levels of FBG, LDL-C, and TG were measured by the colorimetric kits from Pars Azmoon Trading Company made in Iran. The intra-assay coefficient of variation and sensitivity for glucose, LDL-C, and TG were 1 mg/dl.

### Statistical analysis

All results are expressed as mean ± SEM. The normal distribution of data was examined using the Shapiro Wilk test, and for testing the variance congruence, the Levene’s test was used. One-way analysis of variance (Tukey posthoc test) was used to find inter-group differences, and repeated measures ANOVA (Bonferroni posthoc test) was used to find within-group differences. Pearson correlation test was used to examine the relationship between body weight (BW), leptin, and TNF-α levels with the studied variables in a stage of HFD + STZ, exercise training, and de-training. All statistical analyses were performed on SPSS 22 and the level of p < 0.05 was considered as statistically significant.

## Results

### Body-weight changes, concentration of FBG, insulin, TG, LDL-C, leptin, and TNF-α before and after induction of diabetes in Wistar rats

**Fig. 2:**
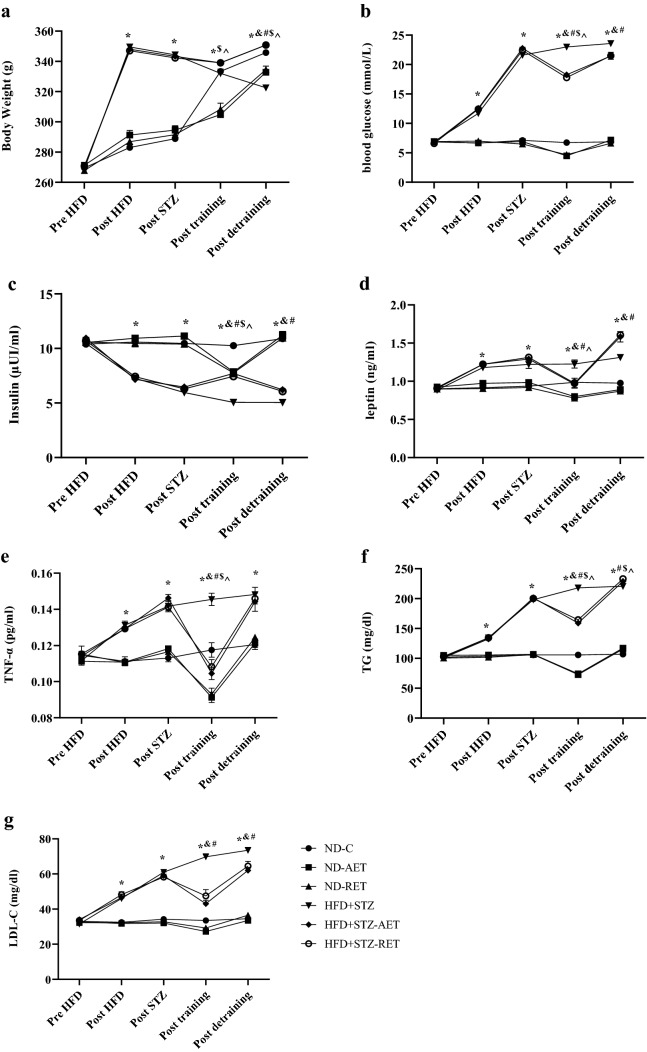
Selected general and biochemical factors in different time of research protocol. Body weight (**a**), fasting blood glucose (**b**), insulin (**c**), leptin (**d**), TNF-α (**e**), TG (**f**), and LDL-C (**g**) levels at baseline, after high-fat diet (HFD), after STZ injection, after 12 weeks of exercise training, and after 4 weeks of de-training is shown. The groups consisted of healthy rats that received a normal diet (ND) (control (ND-C), resistance exercise training (ND-RET), aerobic exercise training (ND-AET)), and low-dose STZ-induced diabetic rats that received a high-fat diet (HFD + STZ) (control (HFD + STZ-C), resistance exercise training (HFD + STZ-RET), and aerobic exercise training (HFD + STZ-AET)). Data are expressed as the mean ± SEM. *p < 0.001 for the HFD + STZ-C group *vs*. ND-C group, ^&^p < 0.01 for the HFD + STZ-C group vs. HFD + STZ-AET group, ^#^p < 0.01 for the HFD + STZ-C group vs. the HFD + STZ-RET group, ^$^p < 0.01 for the ND-C group vs. the ND-AET group, and **^**p < 0.01 for the ND-C group vs. the ND-RET group

Wistar rats had a mean body weight of 269.68 ± 3.29 g on the first day of the study (p > 0.05). Consumption of HFD along with a single low-dose STZ injection for 4 weeks significantly increased BW compared to the beginning stage (p < 0.001). Comparing the groups after 4 weeks of HFD + STZ showed that the BW increase of HFD + STZ rats’ groups was significantly higher than that of ND rats’ groups (p < 0.001). Besides, 4 weeks of HFD + STZ significantly increased FBG (p < 0.001), and decreased serum insulin (p < 0.001) relative to the beginning stage, and the difference between the ND and HFD + STZ groups in FBG and serum insulin was statistically significant (p < 0.001). Also, this period increased TG, LDL-C, leptin, and TNF-α levels in HFD + STZ rats’ groups compared to the beginning stage (p < 0.05), and ND rats’ groups (p < 0.001). The changes in BW, FBG, and serum insulin induced by HFD + STZ confirmed the induction of type 2 diabetes (see Fig. 2).

### Changes in BW, FBG, insulin, TG, LDL-C, leptin, and TNF-α concentrations after 12 weeks of RET and AET in HFD + STZ and ND groups

Twelve weeks of RET and AET in the HFD + STZ-RET, HFD + STZ-AET groups led to a significant decrease in TG, LDL-C, leptin, TNF-α, FBG levels, and a significant increase in insulin compared to HFD + STZ-C (p < 0.01). There was no difference in BW between the HFD + STZ-RET, HFD + STZ-AET groups, and HFD + STZ-C groups (p > 0.05). There was no difference between HFD + STZ-RET, HFD + STZ-AET groups in the studied variables (p > 0.05). Also, 12 weeks of RET and AET in the ND-RET, ND-AET groups led to a significant decrease in BW, TG, TNF-α, FBG, and insulin levels compared to ND-C (p < 0.01). Only the RET program in the ND-RET group resulted in a significant decrease in leptin compared to ND-C (p < 0.05). There was no difference in LDL-C between the ND-RET, ND-AET groups, and ND-C groups (p > 0.05). There was no difference between ND-RET, ND-AET groups in the studied variables (p > 0.05). Comparison of the HFD + STZ-RET and HFD + STZ-AET groups with the ND-C group after 12 weeks of RET and AET showed that there was no difference in leptin and TNF-α levels (p > 0.05) (see Fig. 2).

### Changes in BW, FBG, insulin, leptin, and TNF-α concentration after 4 weeks of de-training in the all trained groups

Four weeks of de-training in the HFD + STZ-RET, HFD + STZ-AET groups led to a significant increase in BW, TG, LDL-C, leptin, TNF-α, and FBG compared to end of the exercise training stage (p < 0.05), but the reduction of insulin in HFD + STZ-RET, HFD + STZ-AET groups was not significant compared to end of the exercise training stage (p > 0.05). Besides, 4 weeks of de-training led to in the ND-RET and ND-AET groups a significant increase in BW, TG, leptin, TNF-α compared to the end of the exercise training stage (p < 0.05). Also, an increase in FBG level in ND-AET group, and a decrease in insulin level in ND-RET group compared to the end of the exercise training a stage were observed (p < 0.05), and an increase of LDL-C in the ND-RET and ND-AET groups was not significant compared to the end of the exercise training stage (p > 0.05; see Fig. 2).

### The correlation between body weight, leptin, and TNF-α levels with studied variables in stage of HFD + STZ, exercise training, and de-training

Pearson’s correlation test in the HFD + STZ stage showed, BW had a very high positive relationship with levels of leptin, TNF-α, FBG, TG, and LDL-C, and a very high negative relationship with insulin levels (p < 0.001, 0.90 < r < 1). Following 12 weeks of RET and AET, the correlation between BW and the studied variables decreased compared to the HFD + STZ stage, so that, the relationship between BW with leptin, TNF-α, and LDL-C was moderate and positive (p < 0.01, 0.50 < r < 0.69), and it had a positive and high relationship with FBG and TG (p < 0.001, 0.70 < r < 0.89). Also, there was no significant relationship between BW and insulin after 12 weeks of exercise training. In the de-training period, there was only a small and positive correlation between BW and leptin (p < 0.05, 0.26 < r < 0.49) (Table 2).

**Table 2:** The correlation between body weights with the studied variables

Body weight	Leptin	TNF-α	FBG	Insulin	TG	LDL
HFD + STZ						
Pearson correlation	.934***	.939***	.976***	− .949***	.975***	.965***
Sig. (2-tailed)	.000	.000	.000	.000	.000	.000
Exercise training						
Pearson correlation	.561**	.522**	.719***	− .004	.723***	.552**
Sig. (2-tailed)	.004	.009	.000	.987	.000	.005
Detraining						
Pearson correlation	.462*	.082	.089	− .073	.200	− .015
Sig. (2-tailed)	.023	.704	.681	.734	.349	.944

Correlation is significant at ***p < 0.001, **p < 0.01, and *p < 0.05

The results of Pearson’s correlation test in the HFD + STZ stage showed that leptin levels had a very high positive relationship with levels of FBG, TG, and LDL-C (p < 0.001, 0.90 < r < 1), a very high negative relationship with insulin levels (p < 0.001, 0.90 < r < 1), and a high positive relationship with TNF-α levels (p < 0.001, 0.70 < r < 0.89). The relationship between leptin with the variables studied decreased after 12 weeks of RET and AET compared to the HFD + STZ stage, so that, the correlation of leptin with TNF-α, LDL-C, FBG, and TG was positive and high (p < 0.001, 0.70 < r < 0.89), and a low negative correlation with insulin (p < 0.05, 0.26 < r < 0.49) was observed after 12 weeks of exercise training. In the de-training stage, leptin correlation with TNF-α, LDL-C, FBG was positive and high (p < 0.001, 0.70 < r < 0.89), and leptin was a very high positive relationship with TG (p < 0.001, 0.90 < r < 1). Leptin-insulin correlation increased during the de-training stage compared to the exercise training stage (p < 0.001, 0.70 < r < 0.89) (Table 3).

**Table 3:** The correlation between leptin with the studied variables

Leptin	TNF-α	FBG	Insulin	BW	TG	LDL
HFD + STZ						
Pearson correlation	.885***	.946***	− .903***	.934***	.953***	.917***
Sig. (2-tailed)	.000	.000	.000	.000	.000	.000
Exercise training						
Pearson correlation	.820***	.735***	− .407*	.561**	.827***	.795***
Sig. (2-tailed)	.000	.000	.048	.004	.000	.000
Detraining						
Pearson correlation	.791***	.892***	− .866***	.462*	.937***	.841***
Sig. (2-tailed)	.000	.000	.000	.023	.000	.000

Correlation is significant at *** p < 0.001, **p < 0.01, and *p < 0.05

Pearson’s correlation test in the HFD + STZ stage showed, TNF-α level had a very high positive relationship with levels of FBG, TG, and LDL-C (p < 0.001, 0.90 < r < 1), and a very high negative relationship with insulin levels (p < 0.001, 0.90 < r < 1). The relationship between TNF-α with the variables studied decreased after 12 weeks of RET and AET compared to the HFD + STZ stage, so that, the correlation of TNF-α with LDL-C and TG was positive and high (p < 0.001, 0.70 < r < 0.89), a moderate and positive correlation with FBG (p < 0.01, 0.50 < r < 0.69), and a low negative correlation with insulin (p < 0.05, 0.26 < r < 0.49) was observed after 12 weeks of exercise training. In the non-exercise stage, TNF-α was highly correlated with all variables except BW (p < 0.001, 0.70 < r < 0.89) (Table 4).

**Table 4:** The correlation between TNF-α with the studied variables

TNF-α	Leptin	FBG	Insulin	BW	TG	LDL
HFD + STZ						
Pearson correlation	.885***	.950***	− .920***	.939***	.952***	.932***
Sig. (2-tailed)	.000	.000	.000	.000	.000	.000
Exercise training						
Pearson correlation	.820***	.670***	− .450*	.522**	.780***	.814***
Sig. (2-tailed)	.000	.000	.027	.009	.000	.000
Detraining						
Pearson correlation	.791***	.884***	− .884***	.082	.893***	.865***
Sig. (2-tailed)	.000	.000	.000	.704	.000	.000

Correlation is significant at ***p < 0.001, **p < 0.01, and *p < 0.05

## Discussion

The results of this study showed that diabetes induction increased BW, FBG, TG, LDL-C, leptin, and TNF-α levels, and decreased insulin compared to the beginning stage. Afterward, 12 weeks of AET and RET significantly decreased leptin, TNF-α, TG, LDL-C, and FBG in all trained groups. We observed a high correlation between BW of type 2 diabetic rats and FBG, TG, LDL-C, leptin, insulin, and TNF-α.

In obesity, the storage capacity of subcutaneous adipose tissue, the largest white adipose tissue depot, is limited, and further caloric overload leads to fat accumulation in ectopic tissues (e.g., liver, skeletal muscle, and heart) and in the visceral adipose depots, an event commonly defined as “lip toxicity.” Excessive ectopic lipid accumulation leads to local inflammation and insulin resistance. Indeed, over-nutrition triggers uncontrolled inflammatory responses in the white adipose tissue leading to chronic low-grade inflammation, thus fostering the progression of insulin resistance [27]. Also, Leptin and TNF-α are involved in the pathogenesis of obesity and insulin resistance [28]. The results of studies showed that leptin concentration correlates positively with BW and Body mass index (BMI) in type 2 diabetes condition [29, 30]; our results were consistent with the findings of these studies. Insulin resistance is often associated with obesity and hyperleptinemia and leads to increased expression of the obesogenic gene and increased leptin level [31]. Our results confirm that there is a high correlation between leptin, TNF-α, and insulin and these correlations cause many disorders in people with diabetes. Plasma leptin levels are directly related to body fat stores and respond to changes in the body’s energy exchange. The role of leptin in obesity was initially thought to be an anti-obesity hormone, but this role is usually reduced by LR, which is one of the causes of obesity [32]. The loss of leptin function and its consequences for the maintenance of energy balance have been well characterized using the Lepob/ob mouse, which serves as a naturally occurring animal model for this metabolic condition [33].

The potential for exercise training in reducing adipose and LRs has been confirmed [34]. Our results showed that 12 weeks of exercise training reduced leptin, TNF-α, and lipid profile, but 4 weeks of de-training caused these adaptations to be lost. So, a short-term de-training was sufficient to return the values of the metabolic variables to the pre-exercise training levels. There was no difference between AET and RET in improving the variables studied. Several studies have examined the effects of exercise training on serum leptin concentrations [34, 35], but a limited number of studies have compared the effects of training and de-training on leptin and TNF-α levels. Abbenhardt et al. [35] reported that aerobic exercise (consisting of 45 min, 60 min/day, 5 days/week, for 12 months), compared to high-intensity exercise, decreased both BW and leptin concentrations and improved hypothalamic leptin sensitivity in women. In another study, leptin level decreased significantly following a 6-month resistance training [36] and a 5-month aerobic training program [37]. To the authors’ knowledge the choice of any of the practice methods can be a good way to reduce leptin. One of the main findings of this study was that 4 weeks of de-training increased the levels of leptin and TNF-ɑ in the diabetic trained groups compared to the diabetic control group. Many studies have confirmed that de-training causes an increase in adiposity, insulin resistance and adipokines secretion and a decrease in exercise performance in non-athletes and athletes [38, 39]. Regarding the relationship between insulin and leptin, the most important and the first hormone causing leptin changes is the insulin hormone. In this study, 12 weeks of AET and RET significantly increased insulin in HFD + STZ rats and decreased insulin in ND rats. Also, our results showed a high negative correlation between insulin and leptin. Jang and Joo [40] reported that the plasma leptin level increased in different conditions following 6 and 12 weeks of de-training with significant differences between the trained and control groups. The possible mechanism involved in the relationship between insulin and leptin is that leptin affects the peripheral resistance of insulin and reduces its activity and signals. Leptin plays an important role in the control of food intake, energy expenditure, metabolism, and body weight. This hormone also has a key function in the regulation of glucose homeostasis. Additionally, β cell mass can be affected by leptin through changes in proliferation, apoptosis, or cell size. All these different functions in the β cell are triggered by leptin as a result of the large diversity of signaling pathways that this hormone is able to activate in the endocrine pancreas. Therefore, leptin can participate in glucose homeostasis owing to different levels of modulation of the pancreatic β cell population [41]. Uysal et al. [42] reported that the treadmill running of rats (for 30 min per session and five times a week for 6 weeks) increased the leptin level in female rats but led to no changes in male rats. The reason for the inconsistency with the findings of this study can be the lower initial weight of the rats and having less body fat, as well as the lower intensity and duration of exercise compared to this study.

In our study, there was a high correlation between TNF-α and TG and LDL-C, which decreased following exercise training and increased after the de-training period. Among pro-inflammatory cytokines, TNF-α plays an important role in inflammation of the tissues, decreasing IRS-1, GLUT4, CEBP-PPAR, perilipin and Acrp30 protein, which leads to T2DM [43]. Lemos et al. [44] reported that regular exercise significantly reduced IL-6 and TNF-α expression in high-fat diabetic rats. Lucotti et al. [45] observed that the TNF-α and monocyte chemoattractant protein-1 (MCP-1) levels significantly decreased in AET but increased in the combined AET and RET group. Also, Aizik et al. [46] showed that following exercise (30 min), the factors in the blood suppress t-cell production of IL-2 and TNF-α. McMahon et al. [47] discovered that 8 weeks of RET did not affect systemic TNFα, and the changes in TNFα levels correlated with muscle loss after 4 weeks of de-training. In the same study, no significant changes occurred in CRP and TNF-α in AET and RET groups in obese men after de-training [48]. It can be stated that the differences may be related to the subjects or the intensity and extent of the exercise. It is proposed that following a resistance exercise in young/healthy participants, circulating TNF-α is not sensitive to resistance exercise-induced changes in muscle size and/or function [47]. Also, the time between the last session of exercise and blood sampling may be a possible reason for the discrepancy in TNF-ɑ level with the mentioned researches. In the studies by McMahon et al. [47] and Nikseresh et al. [48], blood samples were collected 3 to 4 days after the last exercise session, but in our study, sampling was done 48 h after the final training session.

It seems that TNF-α reduces lipid oxidation, decreases HDL-C level, and increases TG and LDL-C levels by increasing the production of FFA and TG, and decreasing endothelial lipoprotein lipase activity [49]. Generally, adipose tissue and inflammation markers are known as key factors in insulin resistance, and we can reduce the level of these indicators by providing a proper training program. According to the findings of this study, both AET and RET methods had an equal impact on improving the risk factors and clinical parameters in the type 2 diabetes model induced by HFD + STZ.

## Conclusions

In summary, the findings indicate that AET and RET can decrease the TG, LDL-C, leptin and TNF-α levels in the diabetic and normal rats after 12 weeks of exercise training. Also, there was no significant difference between the two exercise protocols, and after 4 weeks of de-training, the produced adaptations were lost. Besides, one of the notable findings of this study was the high correlation of the variables examined in three stages including diabetes induction with HFD + STZ, the long-term AET and RET, and the de-training period.

## Data Availability

The primary data for this study is available from the authors on direct request.
